# Gene expression profile changes in the jejunum of weaned piglets after oral administration of *Lactobacillus* or an antibiotic

**DOI:** 10.1038/s41598-017-16158-y

**Published:** 2017-11-17

**Authors:** Dongyan Zhang, Tingting Shang, Yan Huang, Sixin Wang, Hui Liu, Jing Wang, Yamin Wang, Haifeng Ji, Rijun Zhang

**Affiliations:** 1 Laboratory of Feed Biotechnology, State Key Lab. of Animal Nutrition, College of Animal Science & Technology, China Agricultural University, Beijing, 100083 China; 2 Institute of Animal Husbandry and Veterinary Medicine, Beijing Academy of Agriculture and Forestry Sciences, Beijing, 100097 China; 3 College of Information and Electrical Engineering, China Agricultural University, Beijing, 100083 China

## Abstract

The small intestine plays an essential role in the health and well-being of animals. Previous studies have shown that *Lactobacillus* has a protective effect on intestinal morphology, intestinal epithelium integrity and appropriate maturation of gut-associated tissues. Here, gene expression in jejunum tissue of weaned piglets was investigated by RNA-seq analysis after administration of sterile saline, *Lactobacillus reuteri*, or an antibiotic (chlortetracycline). In total, 401 and 293 genes were significantly regulated by chlortetracycline and *L. reuteri*, respectively, compared with control treatment. Notably, the *HP*, *NOX1* and *GPX2* genes were significantly up-regulated in the *L. reuteri* group compared with control, which is related to the antioxidant ability of this strain. In addition, the expression of genes related to arachidonic acid metabolism and linoleic acid metabolism enriched after treatment with *L. reuteri*. The fatty acid composition in the jejunum and colon was examined by GC-MS analysis and suggested that the MUFA C18:1n9c, and PUFAs C18:2n6c and C20:4n6 were increased in the *L. reuteri* group, verifying the GO enrichment and KEGG pathway analyses of the RNA-seq results. The results contribute to our understanding of the probiotic activity of this strain and its application in pig production.

## Introduction

The intestines have various functions such as the absorption of nutrients, the absorption and secretion of electrolytes and water, and the secretion of mucin and immunoglobulins, and they provide a selective barrier against harmful antigens and pathogens^1^. Weaning is one of the most stressful stages in piglet development. During the transition process, piglets must cope with abrupt separation from their mother, a new environment, and a switch from a highly digestible (liquid) milk diet to a less-digestible, more complex, chemically composed solid feed. This process can potentially cause damage to intestinal tissue, resulting in changes in the villus and crypt architecture, and depress the activity of many brush-border digestive enzymes^2^. Consequently, piglets are more susceptible to intestinal complications such as disturbed gut motility, reduced absorptive function, reduced mucosal immune defence, and reduced intestinal regenerative capability, resulting in poor appetite, lower feed intake, and growth retardation^3^. Gastrointestinal disturbances occurring immediately post weaning cause large economic losses in the pig farming industry^4^.

In the recent years, the increased use of antibiotic growth promoters has raised concerns about the development of pathogenic bacterial strains that are resistant to antibiotics and about residual contamination of the food chain with these agents^5^. *Lactobacillus* is part of the normal intestinal flora and is thought to alter the intestinal microbiota, thereby affecting physiological intestinal function and the general health of weaned pigs^6,7^. Studies focusing on intestinal tissues have shown that *Lactobacillus* has a protective effect on tissue structure, the integrity of the intestinal epithelium, appropriate maturation of gut-associated tissues and the function of the neuro-endocrine system^8,9^. Recently, the mechanism by which *Lactobacillus* supports gastrointestinal health in mammals has become of great interest.

With the development of advanced molecular genetics technologies including next-generation sequencing and bioinformatics, transcriptome sequencing (RNA-seq) provides a convenient platform for measuring large-scale gene expression patterns in organisms. RNA-seq also enables the detection of differentially expressed genes (DEGs) with low expression levels^10–14^. Some studies have analysed the transcriptome profiles of blood^15^, muscle^16,17^, gonads^18^, liver^19^, backfat^20^ and uterine endometrium^21^ in pigs, while Mach *et al*.^22^ analysed the gene expression profile along the small intestine and in ileal Peyer’s patches in four young pigs. The above studies have all helped elucidate the mechanisms underlying tissue and organ development and have thus provided valuable information for the pig farming industry. However, studies exploring gene expression in the small intestinal segment in relation to *Lactobacillus* and antibiotic treatments have not been conducted.

The present study aimed to investigate gene expression in jejunum tissue of weaned piglets. RNA-seq gene expression profiling was conducted on weaned piglets that received one of the following treatments: *Lactobacillus reuteri*, chlortetracycline (an antibiotic), or sterile saline (control). The fatty acid composition of the jejunal and colon segments of piglets was also determined to verify the RNA-seq results. The current study should help advance our understanding of *Lactobacillus* and intestinal health and provide basic data for future studies in this area.

## Results

### Sequencing data summary

In this study, sequencing generated approximately 4.82 Gb of raw 150-bp paired-end reads, comprising 1.77, 1.49 and 1.56 Gb from the control, chlortetracycline, and *Lactobacillus* groups, respectively. Using the TopHat2 aligner, over 97.82% of the clean reads per sample were mapped back to the *Sus scrofa* reference genome (Sscrofa v10.2) sequence^23^, the vast majority of the mapped reads were located in annotated exons (Table 1).

**Table 1:** Summary statistics of sequence quality and alignment information for the three treatment groups.

Sample name	control			chlortetracycline			*Lactobacillus reuteri* ZLR003		
	Z1	Z2	Z3	Z4	Z5	Z6	Z7	Z8	Z9
Total reads	48,520,686	56,980,778	71,214,606	51,438,692	47,164,962	50,083,776	52,313,232	52,976,886	50,463,832
Clean reads	47,217,636	55,431,926	69,284,246	49,929,070	45,816,326	48,597,036	50,590,698	51,615,850	49,008,652
Q20, %	96.16	96.11	96.16	96.03	95.97	96.00	95.58	96.31	95.99
Q30, %	90.70	90.57	90.70	90.47	90.33	90.43	89.55	90.99	90.32
GC content, %	53.06	53.27	53.39	53.47	52.91	52.76	53.23	52.42	52.67
Mapping rate, %	79.82	78.14	79.47	77.93	79.92	79.23	79.20	80.53	78.72

Q20 and Q30 represent the proportion of bases with a Phred quality score greater than 20 or 30, respectively.

### Identification and analysis of DEGs among the three groups

Based on the FPKM values, approximately 93% and a little more than 1.3% of the reference genes were expressed at less than 100 FPKM and more than 500 FPKM. The first and second Principal Component Analysis (PCA) was performed and the result showed the different expression patterns among the three treatments (Supplementary Figure S1). To judge the statistical significance of the DEGs, *P*-values ≤ 0.05 and absolute values of |logFC| ≥ 1 were used as the thresholds. In total, 11 genes were differentially expressed among the three groups. 401 DEGs were identified in the chlortetracycline vs. control analysis: 240 up-regulated and 161 down-regulated. Furthermore, 293 DEGs were identified in the *L. reuteri* vs. control analysis: 128 up-regulated and 165 down-regulated. 99 genes were differentially expressed in both the chlortetracycline and *L. reuteri* groups.

### GO and KEGG pathway analysis

**Figure 1:**
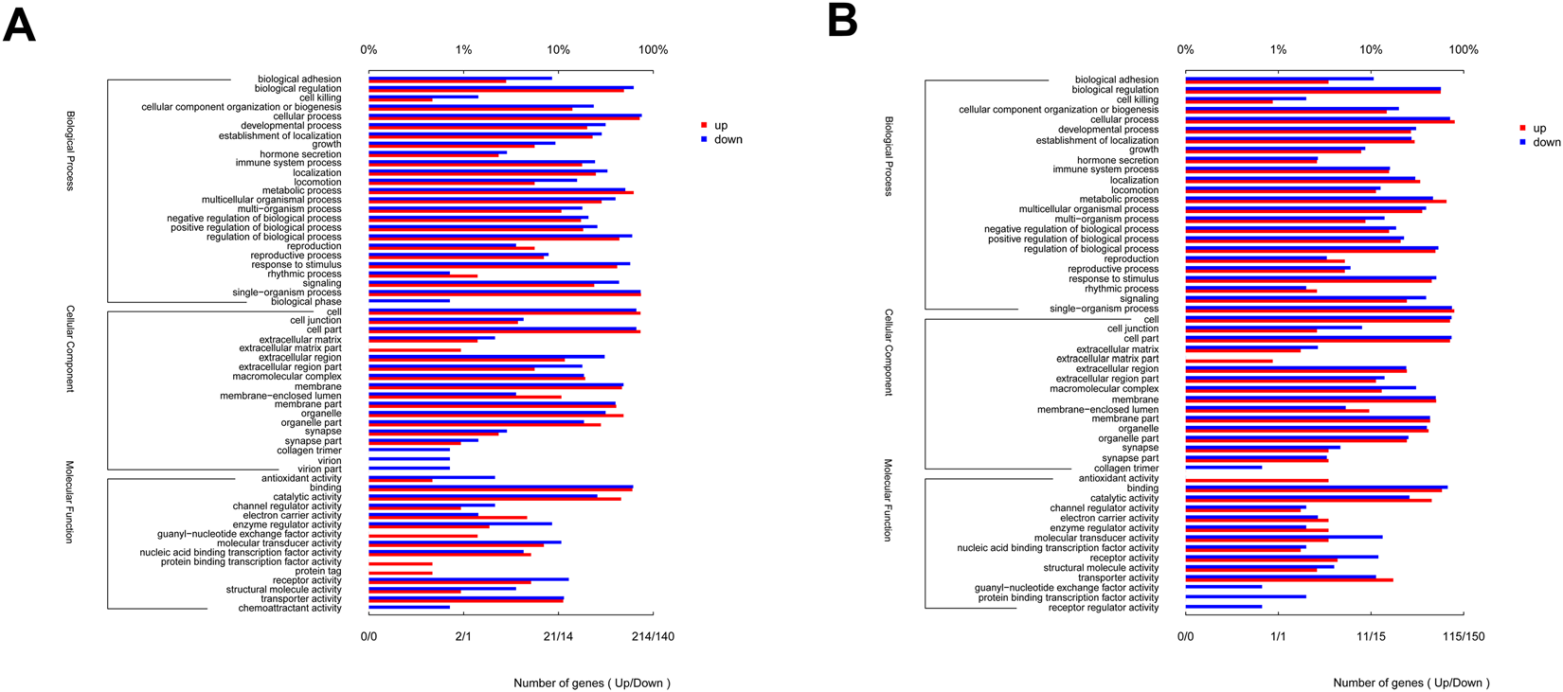
Comparison of gene expression levels among the three treatment groups. (**A**) Jejunum samples in the chlortetracycline vs. control groups. (**B**) Jejunum samples in the *L. reuteri* vs. control groups.

In total, 1374 and 388 DEGs had KEGG pathway annotations in the chlortetracycline vs. control, and *L. reuteri* vs. control groups, respectively. Gene ontology (GO) analysis was performed to investigate which biological functions are important after treatment. As shown in Fig. 1A,B, the GO analysis was classified into the following three functional categories: biological process, cellular component and molecular function, which comprise 25, 18 and 25 for chlortetracycline vs. control, and 24, 16, and 14 for *L. reuteri* vs. control. The results also shown that genes related to the GO terms “Antioxidant activity” and “Receptor regulator activity” were significantly expressed in the *L. reuteri* group compared with the control group. Furthermore, genes related to the GO term “Extracellular matrix part” were significantly expressed by chlortetracycline and *L. reuteri* compared with control.

**Figure 2:**
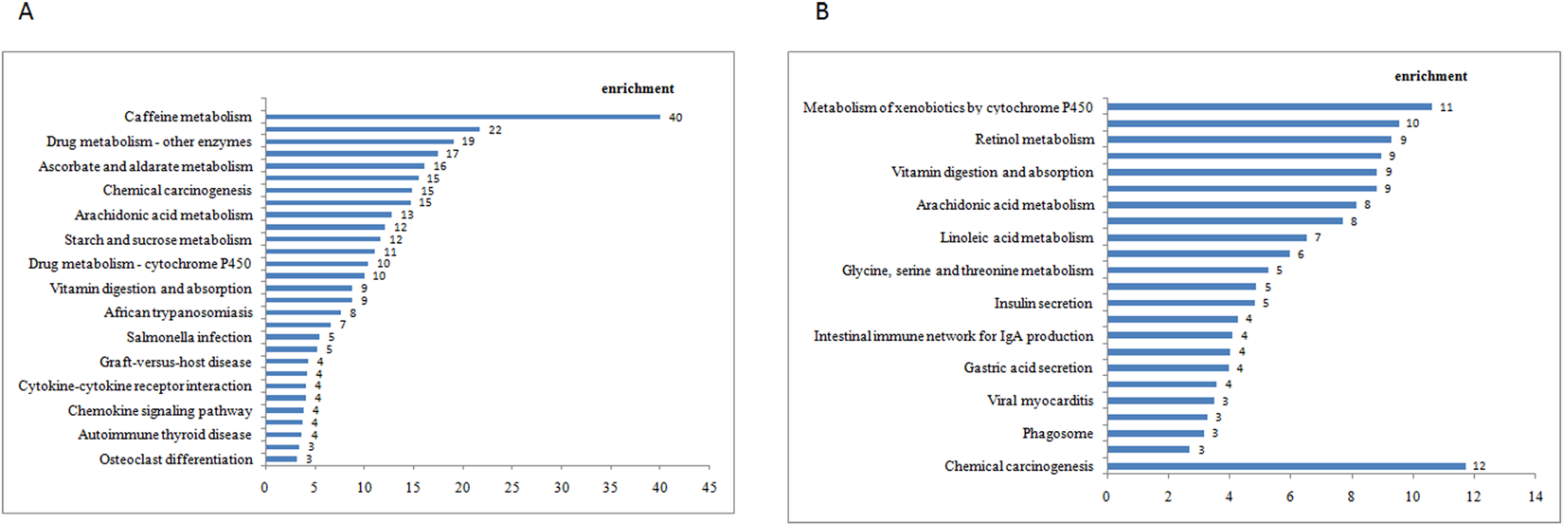
Significantly enriched DEG pathways among the three treatment groups. (**A**) Jejunum samples from the chlortetracycline vs. control groups. (**B**) Jejunum samples from the *L. reuteri* vs. control groups.

Kyoto Encyclopedia of Genes and Genomes (KEGG) pathway analysis was performed to predict the significantly enriched metabolic and immune pathways listed in Fig. 2A,B. In total, 1083 and 991 GO enrichment terms and 78 KEGG enrichment pathways were shared by the chlortetracycline and *L. reuteri* groups, and 30 and 23 KEGG pathways were significantly enriched by both treatments, respectively.

### qRT-PCR confirmation

Using the same RNA samples, the fold change (2^−ΔΔCt^) in the expression of eight genes was determined using qRT-PCR to validate the RNA-seq results. The expression patterns of the eight genes were generally consistent with the RNA-seq results, suggesting that the RNA-seq results were accurate and reliable (Tables 2 and 3).

**Table 2:** List of primers used for qRT-PCR.

Gene	Description	Primer sequence
*DDX3X*	PREDICTED: ATP-dependent RNA helicase DDX3X-like isoform 1	F: AGTTGAACAAGATACTATGCCACR: GAGCCAACTCTACCTACAGC
*TBXAS1*	thromboxane-A synthase	F: ATTTTGCCCAATAAGAAGCGAGAR: ATGTCGCAAATCCAGAACCAT
*ACACB*	acetyl-CoA carboxylase 2 precursor	F: AAGTTTGGAGCCTACATCGTR: ATATTTCTATGCACAGCGGGTT
*LTF*	lactotransferrin	F: GAAATGCGTATCCCAACCTGTR: AAAAGCCACATCTCCAATCCC
*SAA3*	serum amyloid A3 precursor [Sus serum amyloid A3]	F: AAGACATGTGGAGAGCCTACTCGR: TCTCTGGCATCGCTGATCACT
*HP*	haptoglobin precursor	F: CCATCCTGACAACTCCACGGTAR: CAGACACGTAGCCCACAAGC
*SIGLEC1*	sialoadhesin	F: CGAGCCTCCTCTTCCTACTGTTGR: CATCTGCGTGGTTTCCTTCCGA
*CYP2C49*	cytochrome P450 2C49 precursor [Sus cytochrome P450 2C49]	F: AGTCTATGGCCCTGTATTCACCR: AACTCTCTCAGCCATTGGGAA
*ACTB*		F: GGACCTGACCGACTACCTCR: CATCTCCTGCTCGAAGTCCA

**Table 3:** Validation of select RNA-seq-based gene expression data by qRT-PCR analysis.

Gene	A _vs_ C					LR _vs_ C				
	RNA-seq	*P*-value	qRT-PCR	*P*-value	R^2^	RNA-seq	*P*-value	qRT-PCR	*P*-value	R^2^
*DDX3X*	0.65	0.999465	0.24	0.152	0.939	−7.82	0.010325	−0.56	0.021	0.998
*TBXAS1*	−0.56	0.010325	−0.45	0.011	0.703	3.40	0.010325	0.40	0.044	0.995
*ACACB*	0.02	0.999465	−0.07	0.108	0.825	−1.48	0.016843	−1.70	0.049	0.866
*LTF*	0.80	0.999465	0.84	0.153	0.993	2.59	0.010325	2.51	0.003	0.993
*SAA3*	−3.30	0.0340229	−4.88	0.013	0.998	−1.64	0.010325	−1.56	0.004	0.997
*HP*	−0.82	0.562645	−0.85	0.065	0.729	1.23	0.023056	1.97	0.044	0.563
*SIGLEC1*	0.73	0.0340229	0.54	0.016	0.535	−1.60	0.010325	−2.23	0.001	0.999
*CYP2C49*	1.42	0.010325	2.17	0.002	0.965	−3.26	0.010325	−3.76	0.001	0.998

Positive and negative values indicate up- or down-regulation in the comparisons. RNA-seq data are shown as log2 ratios, and qRT-PCR data were calculated by the 2^−ΔΔCt^ method (Mao *et al*., 2013; Kanehisa *et al*., 2008) with *ACTB* as an internal control. R^2^ means Pearson’s Correlation between RNA-seq and qRT-PCR data.

### GC-MS analysis of the fatty acid composition of the jejunum and colon

The profiles of fatty acids, including saturated fatty acids (SFAs), monounsaturated fatty acids (MUFAs) and polyunsaturated fatty acids (PUFAs), were determined in the jejunum and colon of weaned piglets in the three treatment groups. In the jejunum, the SFAs C12:0, C16:0, C17:0, C18:0, C20:0, C22:0 and C24:0 were decreased by *L. reuteri* treatment. The MUFA C18:1n9c, and the PUFAs C18:2n6c and C20:4n6 were lower in the *L. reuteri* group than in the chlortetracycline group (Table 4). However, in the colon, *L. reuteri* treatment decreased the levels of the SFAs C16:0, C17:0, C18:0, and C20:0, whereas MUFA C18:1n9c, PUFAs C18:2n6c, C18:3n3 and C20:4n6 were significantly increased by supplementation with *L. reuteri* (Table 5).

**Table 4:** Fatty acid composition in the jejunal segment of weaned piglets among the three treatment groups.

Fatty acid composition (mg/g)	control	chlortetracycline	*L. reuteri*	*P-value*	SEM^A^
**Saturated fatty acids (SFAs)**					
C12:0	0.219	0.624	0.188	0.051	0.106
C14:0	0.260	0.106	0.120	0.068	0.065
C15:0	0.066	0.109	0.054	0.056	0.026
C16:0	10.055	25.486	5.598	0.005	3.866
C17:0	0.158	0.217	0.046	0.047	0.034
C18:0	4.434	8.818	2.123	0.023	1.213
C20:0	0.251	0.637	0.162	0.041	0.271
C21:0	0.138	0.206	0.079	0.065	0.026
C22:0	0.266	0.581	0.094	0.049	0.097
C24:0	0.415	0.960	0.242	0.025	0.143
**Monounsaturated fatty acids (MUFAs)**					
C16:1	0.312	0.753	0.272	0.085	0.124
C18:1n9c	7.678	15.917	2.717	0.017	2.521
C20:1	0.242	0.202	0.010	0.065	0.050
C22:1n9	0.038	0.015	0.010	0.074	0.006
C24:1	0.223	0.319	0.128	0.150	0.041
**Polyunsaturated fatty acids (PUFAs)**					
C18:2n6c	10.216	22.868	8.317	0.014	3.732
C18:3n3	0.581	0.419	0.107	0.112	0.100
C20:3n6	0.093	0.027	0.010	0.072	0.017
C20:4n6	0.745	2.839	0.867	0.044	0.458
C22:2	0.019	0.018	0.010	0.065	0.004
C22:6n3	0.325	0.852	0.564	0.133	0.012

^A^SEM = standard error of the mean. Means with different superscripts in the same row are significantly different (*P < *0.05).

**Table 5:** Fatty acid composition in the colon segment of weaned piglets among the three treatment groups.

Fatty acid composition (mg/g)	control	chlortetracycline	*L. reuteri*	*P-value*	SEM^A^
**Saturated fatty acids (SFAs)**					
C12:0	0.385	0.275	0.436	0.801	0.076
C14:0	0.878	0.865	0.382	0.091	0.118
C15:0	0.021	0.031	0.040	0.846	0.012
C16:0	22.789	32.704	26.651	0.023	2.863
C17:0	1.159	0.597	0.318	0.041	0.159
C18:0	28.853	51.600	10.345	0.032	7.712
C20:0	1.536	4.308	0.908	0.015	0.630
C21:0	0.273	0.139	0.171	0.195	0.051
C22:0	1.058	1.522	0.792	0.280	0.197
C24:0	1.082	1.520	0.684	0.103	0.172
**Monounsaturated fatty acids (MUFAs)**					
C16:1	0.254	0.116	0.125	0.330	0.034
C18:1n9c	5.328	4.650	15.137	0.024	2.233
C20:1	0.252	0.095	0.274	0.055	0.064
C22:1n9	0.104	0.046	0.029	0.062	0.015
C24:1	2.079	0.587	0.062	0.043	0.388
**Polyunsaturated fatty acids (PUFAs)**					
C18:2n6c	4.735	3.040	17.444	0.011	2.910
C18:3n3	0.339	0.337	1.123	0.041	0.178
C20:3n6	0.094	0.030	0.041	0.308	0.016
C20:4n6	0.179	0.099	0.209	0.012	0.019
C22:2	0.214	0.171	0.038	0.059	0.037
C22:6n3	0.791	0.172	0.196	0.039	0.125

^A^SEM = standard error of the mean. Means with different superscripts in the same row are significantly different (*P < *0.05).

## Discussion

The mammalian gut is susceptible to stress in early life, in addition to genetic determinants of intestinal health, mammals must adapt to a variety of environments, diets, and stressors to maintain intestinal health, and the health consequences of forced adaptation in the form of early weaning may persist into later life. The expression of intestinal tract genes is regulated by many factors, and the expression patterns change dynamically during periods of growth, with numerous gene families and classes differentially expressed in different cell populations of the gut^24^. The weaning transition is a complex period, and profound changes occur in the small intestines of piglets undergoing weaning^25^. Therefore, to better understand the cellular physiology and functionality of the small intestine, the present study used RNA-seq technology to evaluate the transcription profiles of jejunum tissue from weaned piglets treated with *L. reuteri*, chlortetracycline, or sterile saline (control).

The small intestine is mainly responsible for digestion and absorption, and the jejunal segment participates in the maintenance of biological processes, cell structure, cellular components and molecular functions of the intestine. Our results clearly showed differential gene expression profiles in jejunum tissue in response to the three treatments. In particular, 11 genes, including C-C motif chemokine 22 precursor (*CCL22*), RNA-binding region containing protein 2-like (*S100A9*), immune-responsive gene 1 protein homolog (*IRG1*), cytochrome P450 2C49 precursor (*CYP2C49*), DNA nucleotidylexotransferase-like (*DNTT*), putative aldo-keto reductase family 1 member C1 (*AKR1C1*), inter-alpha-trypsin inhibitor heavy chain H4 precursor (*ITIH4*), uteroglobin-like (*SCGB1A1*), serum amyloid A2 precursor (*SAA4*), serpin A3-8 (*ENSSSCG00000030371*), and immune-responsive gene 1 protein homolog (*ENSSSCG00000030557*), were differentially expressed among the three groups. The *CYP2C49* gene is associated with lipid metabolism, as coconut oil and beef tallow increased its expression levels, while backfat subcutaneous adipose tissue showed increased SFAs and decreased MUFAs and PUFAs^26^. Our study showed that *CYP2C49* was down-regulated in the *L. reuteri* group, which may be associated with the fatty acid composition in the intestine. Kim *et al*.^21^ reported that *CYP* genes are also expressed in the uterine endometrial tissues of pigs during pregnancy, with levels increasing throughout gestation and declining at the end of pregnancy (day 114). The other genes, such as *CCL22*, *S100A9*, *SAA4*, and *SCGB1A1*, have been shown to be associated with immunomodulation^27–30^. *AKR1C1* has crucial roles in the biosynthesis and inactivation of all classes of steroid hormones^31^. The above genes have been studied mainly in humans, rats and cell lines *in vitro*. Our study showed that *AKR1C1* was up-regulated, *SCGB1A1* was down-regulated, and *CCL22* and *S100A9* were moderately regulated in the *L. reuteri* group. Previous studies have suggested that the *Lactobacillus* genus involved in pro-inflammatory NF-κB signalling, which TLR4 and NOD2 receptors were down-regulated in rat jejunum^32^. Therefore, the correlation between gene expression and immune signalling pathways affected by *L. reuteri* in piglet jejunum needs to be studied in the future.

In addition, the DEGs in our study were mainly classified into two important categories according to function: nutrient metabolism and immunoregulation. We detected DEGs associated with nutrient metabolism, including mineral, vitamin, amino acid and lipid metabolism, such as the UDP glucuronosyltransferase 2 family, polypeptide A3 (*UGT2A3*), lactotransferrin (*LTF*), and calmegin precursor (*CLGN*), in the *L. reuteri* group compared with the control group, solute carrier family 2 (*SLC2A8*), S100 calcium binding protein A9 (*S100A9*), fatty acid desaturase 3 (*FADS3*), alanine-glyoxylate aminotransferase 2 (*AGXT2*) and vitamin D3 receptor (*VDR*) were differentially expressed in the chlortetracycline group compared with the control group. The DEGs associated with immunoregulation included immunoglobulin kappa variable region (*IGKV-3*), which was differentially expressed in the *L. reuteri* group compared with the control group, and T-cell immunoglobulin and mucin domain containing 4 (*TIMD4*) and CD79b molecule (*CD79B*), which were differentially expressed in the chlortetracycline group compared with the control group.

Mach *et al*.^22^ confirmed that sucrose-isomaltase (*SI*), dipeptidyl peptidase (*DPP4*), and enterocyte sodium/glucose co-transporter (*SLC5A1*) were significantly expressed in jejunum tissue of Large White male pigs at 70 days of age. Our results indicated that *SI*, *DPP4*, and *SLC5A1* were expressed in the jejunal segment of weaning piglets in all three treatment groups. However, *SI* gene expression was significantly higher in the chlortetracycline group than in the control group, while it showed an increasing trend (P = 0.062) in the *L. reuteri* group compared with the control group. *SI* is the only enzyme reported to hydrolyse sucrose in the small intestine, and it plays a pivotal role in the digestion and absorption of carbohydrates^33^. The differences in *SI* expression in our present study suggested differences in carbohydrate metabolism in response to chlortetracycline treatment. Our previous study indicated that *Proteobacteria* were significantly more abundant in the chlortetracycline and *L. reuteri* groups. However, the increase in the relative abundance of *Proteobacteria* was mostly correlated with an increase in the relative abundance of *Actinobacillus* in the chlortetracycline group and with increases in the relative abundance of *Actinobacillus* and *Escherichia-Shigella* in the *L. reuteri* group^34^. Therefore, the results may be explained by changes in the composition of gut microbiota after oral administration of chlortetracycline or *Lactobacillus* in weaned piglets.

We investigated the biological functions of the DEGs by GO annotation and KEGG pathway analysis. The GO term “Antioxidant activity” was clearly enriched by *L. reuteri* treatment. Notably, haptoglobin precursor (*HP*) and *NADPH* oxidase 1 were significantly expressed in the *L. reuteri* group. Our previous studies suggested that *Lactobacillus* strains increase the serum concentrations of superoxide dismutase, glutathione peroxidase and catalase in weaned piglets^35^. Kumar *et al*.^36^ observed that the activity of catalase, superoxide dismutase and glutathione S-transferase (GST) increased in animals treated with probiotics. It is believed that probiotics can increase GST activity through the action of butyric acid. This short chain fatty acid can change the histone acetylation status, thus increasing the expression of GST^37^. In addition, the GO term “Receptor regulator activity” was also enriched by *L. reuteri* treatment. Thus, we hypothesised that this *Lactobacillus* strain can improve oxidative stress and homeostasis in the piglet intestine.

To investigate the biological functions of the DEGs, we performed GO enrichment and KEGG pathway analyses. Our data clearly revealed that the expression of genes related to retinol metabolism, arachidonic acid metabolism, linoleic acid metabolism and the chemokine signalling pathway increased after treatment with *L. reuteri* treatment. *L. reuteri* treatment also increased the expression of genes related to mineral absorption, cytokine-cytokine receptor interaction, vitamin digestion and absorption, intestinal immune network for IgA production, glutathione metabolism, glycine, and serine and threonine metabolism compared with control treatment. However, retinol metabolism, linoleic acid metabolism, arachidonic acid metabolism, starch and sucrose metabolism, cytokine-cytokine receptor interaction, ascorbate and aldarate metabolism, pentose and glucuronate interconversion, glycine, serine and threonine metabolism, and mineral absorption were induced by chlortetracycline treatment compared with control treatment. In agreement with our present study on the diversity and composition of the gut microbiota of weaned piglets^33^, the differences we observed in the biological functions of the DEGs among the three treatment groups may be related to the intestinal microbiota composition.

Our current study also showed that seven categories of SFAs in the jejunum and four in the colon were significantly decreased and that UFAs, such as MUFA C18:1n9c, PUFAs C18:2n6c, C18:3n3 and C20:4n6, were significantly increased in the *L. reuteri* group compared with the control group, these results were consistent with the DEGs results, in which *FABP3*, *FABP1* and *FADS3* were up-regulated in the *L. reuteri* group, and with the GO enrichment and KEGG pathway analyses, which identified linoleic acid metabolism and arachidonic acid metabolism as significantly increased in the *L. reuteri* group. Mpofu *et al*.^38^ reported higher SFA levels in breast muscle of broilers in the antibiotic group than in the *Lippia javanica* group. Studies have also suggested that *Lactobacillus* strains can improve the biosynthesis of linoleic acid or conjugated linoleic acid *in vitro* ^39,40^. Swiatkiewicz *et al*.^26^ reported that the presence of lipids in pig tissues and their degree of saturation affect the sensory quality of the meat, while adipose tissue metabolism depends on dietary fatty acid composition and is controlled by changes in gene expression. Therefore, we will conduct more experiments to elucidate the effect of *Lactobacillus* strains on the fatty acid composition of porcine muscle, which is critical for pork quality and human health.

## Conclusion

In summary, the gene expression profiles of jejunum tissue from weaned piglets were distinct in response to the three treatments used in this study (control, chlortetracycline, and *L. reuteri* ZLR003). Our study showed that the *L. reuteri* ZLR003 strain promoted antioxidant activity, which is related to oxidative stress in pig production. In addition, the fatty acid composition in the jejunum and colon was improved in the *L. reuteri* ZLR003 group. This study elucidated the probiotic mechanisms of the *L. reuteri* ZLR003 strain in weaned piglets *in vitro* and *in vivo*. Although more research is required, our findings may lead to the future use of *Lactobacillus* strains to improve the gut health of farmed animals.

## Methods

### Ethical approval

Protocols and procedures related to animal work throughout the study were approved by the Institutional Animal Care and Use Committee (IACUC) of the Institute of Animal Husbandry and Veterinary Medicine, Beijing Academy of Agriculture and Forestry Sciences, China. The approval number is IACUC-2010. All pigs used in this study were taken care and operated according to the relevant regulations.

### *Lactobacillus* and chlortetracycline preparation

The *L. reuteri* ZLR003 strain used in the present study was deposited at the China General Microbiological Culture Collection Center (CGMCC) under CGMCC number 11530. The strain was inoculated in Mann, Rogosa and Sharpe (MRS) broth (Merck, Darmstadt, Germany) with an inoculum dose of 1.0% (v/v) for 18 h at 37 °C. Bacterial cells were harvested by centrifugation at 6738 × *g* for 10 min at 4 °C under aseptic conditions, followed by suspension in a 0.85% sterile saline solution. A liquid preparation containing 2.0 × 10^9^ colony forming units (cfu)/mL of viable cells was used. Chlortetracycline ( > 95% purity, Mellon, Biological Technology Co., Ltd., Dalian, China) was prepared at 100 mg/kg using 0.85% sterile saline solution. A 0.85% sterile saline solution lacking *L. reuteri* ZLR003 or chlortetracycline was used as the control treatment. The three treatments were prepared concurrently every 2 days and stored at 4 °C.

### Animal selection and collection of tissue samples

A total of nine crossbred (Landrace × Large White) piglets weaned at 30 days of age and weighing 8.57 ± 1.28 kg (BW) were used under the same husbandry conditions. Piglets were randomly divided into three groups (four females and five males) with a consistent average weight: 1) control treatment, piglets received 5 mL of 0.85% sterile saline solution orally every morning; 2) *L. reuteri* treatment, piglets were orally administered 5 mL of *L. reuteri* ZLR003 (2.0 × 10^9^ cfu/mL); 3) antibiotic treatment, piglets were orally administered 5 mL of 100 mg/kg chlortetracycline. All piglets were given a specifically designed basic diet did not contain any antibiotics^41^ (Table 6). Piglets were housed in an environmentally controlled stainless steel cage with a room temperature between 25 °C and 28 °C and 60% relative humidity. Feed and water were provided *ad libitum* through a feeder and nipple drinker throughout the experimental period, which lasted 10 d.

**Table 6:** Ingredients and composition of the basal diet.

Ingredients (g/kg)	Content
Corn	600
Soybean meal	230
Wheat bran	50.0
Fish meal	20.0
Whey	50.0
Soybean oil	10.0
Premix	40.0
**Chemical composition**	
Digestible energy^a^, MJ/kg	13.75
Crude protein^b^	190
Lysine	11.7
Methionine	3.5
Salt	4.4
Calcium^b^	8.0
Total phosphorus^b^	6.5

^1^Each kg of complete feed contains the following: vitamin A, 11,000 IU; vitamin D_3_, 2,800 IU; vitamin E, 36 mg; menadione, 2.5 mg; vitamin B_1_, 2.5 mg; vitamin B_2_, 6.6 mg; vitamin B_6_, 3.0 mg; vitamin B_12_, 0.025 mg; niacin, 25 mg; pantothenic acid, 13 mg; biotin, 0.2 mg; Mn, 55 mg; Fe, 120 mg; Zn, 100 mg; Cu, 12 mg; I, 0.50 mg; and Se, 0.30 mg. ^a^Calculated nutrient levels. ^b^Measured nutrient levels.

At the end of the experiment, the nine piglets were euthanised under anaesthesia by exsanguination. The jejunum tissues were sampled at 5 cm, 24 cm and 40 cm from the duodenum-jejunum junction, and the three sites were combined into one sample. A total of nine samples were collected in sterile containers, snap-frozen in liquid nitrogen and stored at −80 °C. The contents of the two intestinal segments (jejunum and colon) were collected simultaneously, and all 18 samples were snap-frozen in sterile containers in liquid nitrogen and stored at –80 °C.

### RNA extraction

Total RNA was extracted from tissues using TRIzol® Reagent according to the manufacturer’s instructions (Invitrogen, Carlsbad, CA, USA), and genomic DNA was removed using DNase I (TaKara, Japan). RNA quality was determined using a 2100 Bioanalyser (Agilent Technologies, Palo Alto, CA, USA) and quantified using a ND-2000 (Nano Drop Technologies, Wilmington, DE, USA). A high-quality RNA sample (OD_260/280_ = 1.8–2.2, OD_260/230_ ≥ 2.0, RIN ≥ 8.0, 28 S:18 S ≥ 1.0, > 10 μg) was used to construct the sequencing library.

### Library preparation and Illumina HiSeq. 4000 sequencing

Total RNA (5 μg) was prepared for the RNA-seq transcriptome library using the TruSeq™ RNA sample preparation kit according to the manufacturer’s instructions (Illumina, San Diego, CA, USA). Thereafter, mRNA was isolated using the polyA selection method with oligo (dT) beads, followed by fragmentation (100 to 400 bp) in fragmentation buffer. Next, double-stranded cDNA was synthesised using a Super Script double-stranded cDNA synthesis kit (Invitrogen) with random hexamer primers (Illumina) and then subjected to end-repair, phosphorylation and ‘A’ base addition according to the Illumina library construction protocol. Nine cDNA libraries from the following three groups, and libraries were size-selected for cDNA target fragments of 200–300 bp on 2% Low Range Ultra Agarose followed by 15 cycles of PCR amplification using Phusion DNA polymerase (NEB, USA). After quantitation by TBS-380 (Tumer BioSystems, Sunnyvale, CA, USA), the paired-end RNA-seq library was sequenced on an Illumina HiSeq. 4000 (2 × 150-bp read lengths). The raw reads were deposited in the National Center for Biotechnology Information Sequence Read Archive database (https://www.ncbi.nlm.nih.gov/sra) under accession number: SRR5124832.

### Bioinformatics analysis

#### Read mapping

The raw paired-end reads were trimmed and quality controlled by SeqPrep (https://github.com/jstjohn/SeqPrep) and Sickle (https://github.com/najoshi/sickle) using the default parameters. The clean reads were separately aligned with the reference genome in orientation mode using TopHat2 software (http://tophat.cbcb.umd.edu/)^42^. The bowtie2 mapping criteria stated that sequencing reads should be uniquely matched to the genome allowing up to two mismatches, without insertions or deletions. Next, the gene region was expanded following the site depths for operon attainment. The whole genome was split into multiple 15-kbp windows that shared 5-kbp of sequence. New transcribed regions were defined as > 2 consecutive windows without an overlapping gene region, where at least two reads mapped per window were in the same orientation.

### Differential expression analysis and functional enrichment among the three groups

To identify DEGs between two different samples, the expression levels of each transcript were calculated using the fragments per kb of exon per million (FPKM) mapped reads method. Cuffdiff (http://cufflinks.cbcb.umd.edu/)^43^ was used for the differential expression analysis. DEGs between two samples were selected using the following criteria: i) the logarithmic fold change was > 2.0, and ii) the false discovery rate was < 0.05. To understand the DEG functions, GO functional enrichment and KEGG pathway analyses were conducted using Goatools (https://github.com/tanghaibao/Goatools) and KOBAS (http://kobas.cbi.pku.edu.cn/home.do)^44^. DEGs were considered significantly enriched in GO terms and metabolic pathways when the Bonferroni-corrected *P*-value was less than 0.05.

### Quantitative real-time PCR (qRT-PCR) confirmation

To confirm the RNA-seq results, eight candidate genes were selected and examined by quantitative reverse transcription PCR (qRT-PCR) in the same RNA samples as were used for RNA-seq. Reverse transcription was performed with 1 μg of extracted RNA in a total volume of 20 μL using TIANScript M-MLV reverse transcriptase (TianGen, Beijing, China) with specific primers (*ACTB*-qPCR-R, *CYP2C49*-qPCR-R, *DDX3X*-qPCR-R, *HP*-qPCR-R, *LTF*-qPCR-R, *SAA3*-qPCR-R, *SIGLEC1*-qPCR-R, *TBXAS1*-qPCR-R and *ACACB*-qPCR-R), according to the manufacturer’s instructions. The resulting cDNA was stored at −20 °C prior to PCR amplification. For qPCR, SYBR Green Real-time PCR Master Mix (TOYOBO, Osaka, Japan) was used to amplify the cDNA using an iQ5 Multicolor Real-Time PCR Detection System (Bio-Rad Laboratories, Hercules, CA, USA). The PCR conditions comprised initial denaturation at 95 °C for 3 min and 40 cycles of 95 °C for 15 s, 60 °C for 30 s, and 72 °C for 20 s. Melting curve data were collected at 0.5 °C increments in 10-s cycles (55 °C for 80 cycles). The gene expression data were normalised against the *ACTB* housekeeping gene data, and the relative expression levels were calculated using the 2^−ΔΔCt^ method. All reactions were conducted on one plate in triplicate to ensure reproducibility.

### GC-MS analysis of the fatty acid composition in the jejunum and colon

The fatty acid composition was determined by gas chromatography (6890 series, Agilent Technologies, Wilmington, DE, USA) according to the procedures of Sukhija and Palmquist^45^ with slight modifications. The samples were converted to fatty acid methyl esters using methanolic HCl. Undecanoic acid (C11:0) was used as the internal standard. Aliquots of 1 μL were injected into a capillary column (60 m × 250 μm × 0.25 μm, DB-23, Agilent) with cyanopropyl methyl silicone as the stationary phase. The column temperature was programmed with a 1:20 split. Injector and detector temperatures were maintained at 260 and 270 °C, respectively. Nitrogen was the carrier gas at a flow rate of 2.0 mL/min.

### Statistical analyses of unsaturated fatty acid data

The fatty acid data were analysed according to the General Linear Models (GLM) procedure in SAS. Differences among means were tested using Tukey’s test, and differences were considered significant at *P* < 0.05.

## Electronic supplementary material

Supplementary Figure S1
