# Supplementary material for: Gene expression profile changes in the jejunum of weaned piglets after oral administration of Lactobacillus or an antibiotic
Source: Sci Rep. 2017 Nov 17;7:15816. doi: 10.1038/s41598-017-16158-y (PMC5693952; doi:10.1038/s41598-017-16158-y)
Supplement: Supplementary file 1 — Supplementary Figure S1 [file 41598_2017_16158_MOESM1_ESM.pdf]

Gene expression profile changes in the jejunum of weaned piglets after oral administration of *Lactobacillus* or an antibiotic

Dongyan Zhang[1,2], Tingting Shang[1], Yan Huang[3], Sixin Wang[2], Hui Liu[2], Jing Wang[2], Yamin Wang[2], Haifeng Ji [2,*], Rijun Zhang[1,*]

[1]Laboratory of Feed Biotechnology, State Key Lab. of Animal Nutrition, College of Animal Science & Technology, China Agricultural University, Beijing, 100083, China. [2]Institute of Animal Husbandry and Veterinary Medicine, Beijing Academy of Agriculture and Forestry Sciences, Beijing, 100097, China. [3]College of Information and Electrical Engineering, China Agricultural University, Beijing, 100083, China.

Correspondence and requests for materials should addressed to Z.R.J. (email: zhangrj621@126.com) or J.H.F. (email: jhf207@126.com).

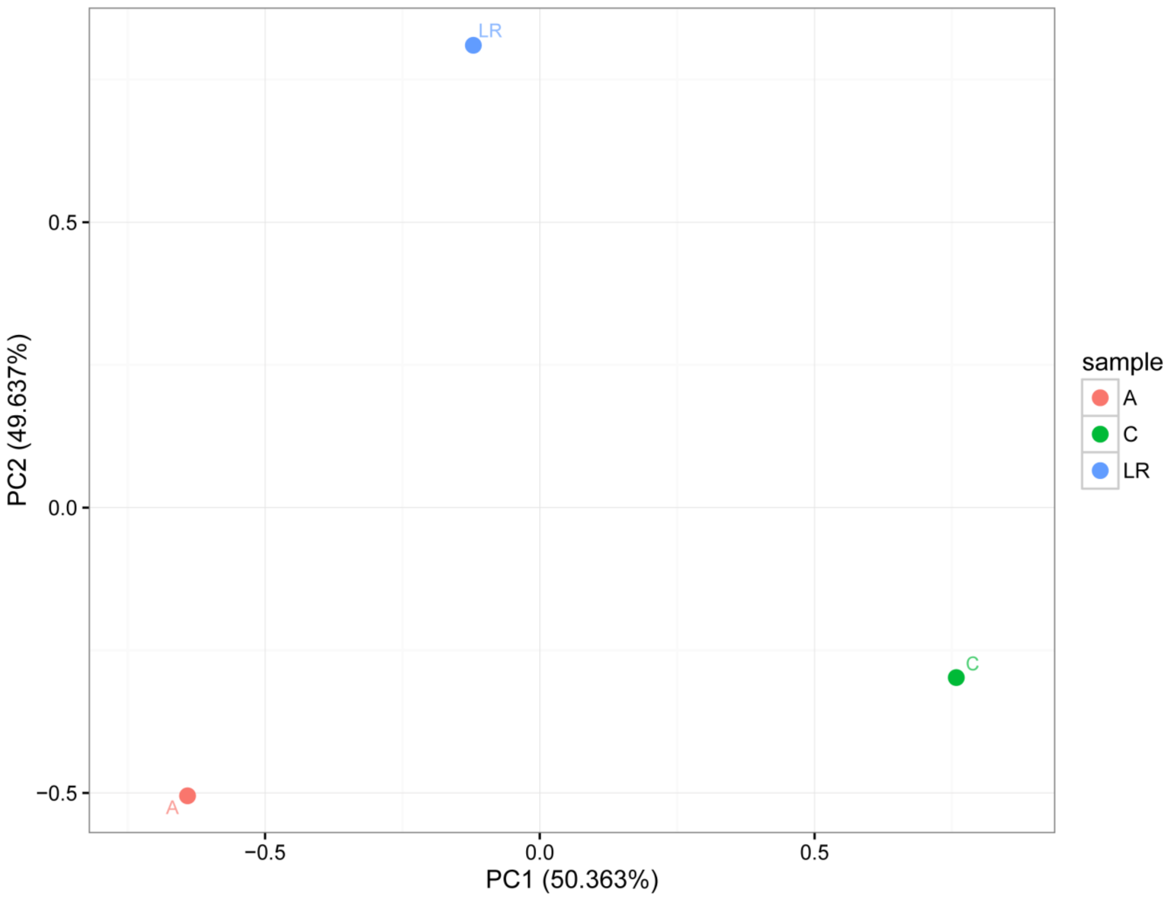

Supplementary Figure 1. Graphical representation of the first and second principal Component Analysis (PCA) among the three treatment groups

C. control group. A. chlortetracycline group. LR. *L. reuteri* group.

Each group was calculated and analyzed by the average mean of three repeats
